# Preoperative monocyte-lymphocyte and neutrophil-lymphocyte but not platelet-lymphocyte ratios are predictive of clinical outcomes in resected patients with non-metastatic Siewert type II/III adenocarcinoma of esophagogastric junction: a prospective cohort study (the AMONP corhort)

**DOI:** 10.18632/oncotarget.15497

**Published:** 2017-02-18

**Authors:** Jia-Wei Zhang, Lei Huang, A-Man Xu

**Affiliations:** ^1^ Department of Gastrointestinal Surgery, The First Affiliated Hospital of Anhui Medical University, Hefei, China; ^2^ German Cancer Research Center (DKFZ), Heidelberg, Germany; ^3^ Department of Gastrointestinal Surgery, The Fourth Affiliated Hospital of Anhui Medical University, Hefei, China

**Keywords:** adenocarcinoma of esophagogastric junction, monocyte-lymphocyte ratio, neutrophil-lymphocyte ratio, platelet-lymphocyte ratio, cancer-specific survival

## Abstract

**Aims:**

To propectively reveal the clinicopathological and prognostic significances of monocyte-lymphocyte ratio (MLR), neutrophil-lymphocyte ratio (NLR) and platelet-lymphocyte ratio (PLR) in resected patients with non-metastatic Siewert type II/III adenocarcinoma of esophagogastric junction (AEG).

**Methods:**

A total of 611 patients diagnosed with Siewert type II/III AEG and undergoing surgery between 2006 and 2011 were prospectively followed-up until April 2016. Associations between preoperative peripheral MLR, NLR, and PLR and clinicopathological parameters were quantified using the multivariate Logistic regression model with adjustment. The correlation between the 3 ratios and cancer-specific survival (CSS) was investigated using the univariate and adjusted multivariate Cox regression models with stratifications. The periodical survival rates for the low- and high-level arms were obtained using the Kaplan-Meier method.

**Results:**

We set the medians (0.223, 2.22, and 124.4) as the cut-off values of preoperative MLR, NLR, and PLR, respectively. MLR was higher in male patients and those > 63 years; PLR was higher in patients with type II tumors. The (marginally-)significantly inverse ratio-CSS association was detected in male patients, those ≤ 63 years, those with type III tumors, and those with pTNM stage III tumors for MLR, and in female patients, those > 63 years, those with type III tumors, those with vessel invasion, and those with stage III tumors for NLR, but was generally negative concerning PLR. The association majorly existed in type III and pTNM stage III tumors.

**Conclusion:**

MLR and NLR might be prognostic factors for patients with non-metastatic Siewert type II/III AEG, while PLR had limited significance.

## INTRODUCTION

The incidence of adenocarcinoma of esophagogastric junction (AEG) has been dramatically increasing in Western countries during the past few decades [1, 2]. Recently, this trend has also occurred in Asia [3]. In China, due to the lack of routine preoperative screening, AEG is often diagnosed at an advanced stage with lymph node metastasis and hematogenous dissemination, resulting in a poor prognosis [3]. In both Western and Eastern countries, AEG-caused mortality is high. According to the location of tumor epicenter, Siewert [4] classified AEG into 3 subgroups. In Western countries, type I AEG (adenocarcinoma of the distal esophagus) is the most prevalent type, and is generally treated as esophageal cancer [5]. In Asia, types II (adenocarcinoma of the cardiac) and III AEG (subcardial adenocarcinoma that infiltrates the esophagogastric junction) are commoner than type I and are mostly treated as gastric cancer [6]. Despite multiple systemic treatment options against AEG, the outcome remains unoptimistic.

There is intense interest in the discovery of prognostic biomarkers that will improve clinical outcomes through risk classification, but most of them have not yet come into routine clinical practice, due to high costs or non-standardization. Although tumor-associated inflammation has not been explicitly elucidated, emerging evidence has revealed that it plays an important role in cancer progression and indicates prognosis [7]. Peripheral blood cells might reflect the inflammatory status of patients. The tumor-related leukocytes, especially monocytes, neutrophils and lymphocytes, which are key regulators of tumor immunity, have essential roles in systemic inflammatory response to malignancies. A series of pretreatment inflammatory biomarkers, including monocyte-lymphocyte ratio (MLR), neutrophil-lymphocyte ratio (NLR) and platelet-lymphocyte ratio (PLR), have been investigated with the hope to predict prognosis in various cancers (*e.g*., esophageal squamous cell carcinoma [8], colorectal cancer [9], and breast cancer [10]). They might be good reflection of hosts’ immune status and tumor burden [11].

However, few studies concerning these inflammatory biomarkers in AEG exist, with the clinical significances and prognostic values remaining obscure. The aim of this study was to investigate the clinical significances and the prognostic values of preoperative MLR, NLR, and PLR for patients with non-metastatic Siewert type II/III AEG.

## MATERIALS AND METHODS

### Patient enrollment and treatment

A total of 641 patients with histologically/cytologically and imaging-diagnostically confirmed non-metastatic Siewert type II/III AEG who required surgical resection were initially consecutively included in this prospective study (the AMONP cohort). All patients were treated at Department of Gastrointestinal Surgery in The First Affiliated Hospital of Anhui Medical University from February 2006 to February 2011. They were in relatively good overall conditions (*e.g*., Hb > 90 g/L, albumin > 30 g/L, good liver and renal function, and ECOG score 0-2), without severe dysfunction of important organs or systematic unfit like dyscrasia, autoimmune or immunodeficiency diseases, infection, or severe mental disorders. They had not received any interventional therapy, cytotoxical treatment, peripheral blood stimulating regimen, or major abdominal surgery before. Patients undergoing multivisceral resection or having other gastrointestinal diseases or malignancies were excluded from this study. Those who had received neoadjuvant therapy or who received blood product transfusion within 1 month before resection were also excluded to avoid the possible influences of such treatments on preoperative laboratory profiles. Inconsistency in diagnosis, pathology, and lesion position before and after resection also excluded the patient from investigation.

All enrolled patients underwent radical surgery. For type II adenocarcinomas invading distal esophagus, transhiatal total gastrectomy (TG) combined with mediastinal lymphadenectomy was conducted. Thoracoabdominal incision might be performed for subtotal esophagectomy to guarantee curability, if the frozen section of proximal esophageal cutting edge was positive even after repeating resection of the distal esophagus. For type III tumors, transabdominal TG was performed. D2 lymphadenectomy was routinely performed. Inferior mediastinal or extended lymph node dissection was performed for patients with esophageal involvement. Intraoperative frozen section was a routine procedure aiming to secure the resection margins free from tumor cells. All operations were conducted by the surgeons who routinely operated on more than 50 cases per year and who had surgical practice of 5 or more consecutive years. After radical surgery, all patients received 4-6 cycles of first-line adjuvant combination chemotherapy with oxaliplatin plus 5-fluorouracil/leucovorin (FOLFOX) or a prodrug of 5-fluorouracil (capecitabine; CapeOX). Patients were excluded if they died of postoperative complications or had positive resection margins.

This study was approved by the Ethics Committee of The First Affiliated Hospital of Anhui Medical University. Written informed consent was obtained from each patient. The study complied was performed in accordance with the guidelines of Declaration of Helsinki [12] and Good Clinical Practice [13].

### Measurement and definition

Preoperatively, gastroscopy, barium meal, computed tomography (CT) and magnetic resonance imaging (MRI) examinations were routinely conducted, forming the basis of AEG Siewert classification and clinical staging [6]. Tumor length, nerve invasion, and vessel involvement were obtained from pathological and surgical reports, providing information for pathological staging. Before analysis, all patients’ tumor stages were (re)assessed according to the 7th version of the TNM staging system by AJCC/UICC [14]. In the current study, cancer-specific survival (CSS) was applied, and was defined as the interval between surgery and AEG-related death/end of follow-up.

### Laboratory test

As part of pretreatment evaluation, all patients’ peripheral blood samples were collected into tubes containing dipotassium ethylenedinitrotetra-acetic acid (EDTA) 3 days before surgery, and all measurements including blood routine tests were performed within 30 minutes after blood collection. MLR, NLR, and PLR were calculated as the ratios of the absolute counts of monocyte, neutrophil, and platelet to lymphocyte, respectively.

### Follow-up

Enrolled patients were prospectively followed-up until April 2016. Follow-up was performed in regular intervals (every 3 months for the first 2 years after treatment, every 6 months in years 3-5, and every 12 months after 5 years). Patients’ evaluations included clinical examination, laboratory tests, and radiological assessment.

### Statistical analysis

The cut-off levels of the 3 ratios were set as the respective medians. Descriptive statistics were applied for the overall patients and those with low- and high-level ratios. The association between clinicopathological features and the 3 ratios in overall patients and those with stages II and III AEG was quantified using the multivariate Logistic regression model with gender, age group (≤ and > 63 years (median)), tumor position, stage, nerve invasion, and vessel involvement adjusted, and with the risk ratio (RR) estimates and the corresponding 95% confidence intervals (CIs) shown. The association of the 3 ratios with CSS was first assessed using both the Cox regression model-based univariate and multivariate analyses applying the continuous ratios, and then in various stratifications of overall patients and the Siewert type-specific subgroups the correlation of high *versus* low level of each ratio in relation to survival was quantified, adjusting gender, age, tumor position, stage, and vessel invasion, with the corresponding survival curves generated. The hazard ratio (HR) estimates with the corresponding 95% CIs were reported. The 3-, 6-, 12-, 24-, 36-, and 60-month survival rate estimates together with the 95% CI for the low- and high-level group of each ratio was obtained using the Kaplan-Meier method. All statistical analyses were performed using R (v. 3.3.1, Vienna, Austria). A two-sided *P* value of < 0.05 was considered statistically significant.

## RESULTS

### Patients’ characteristics

A total of 641 patients were initially enrolled, and 611 cases (390 Siewert type II and 221 type III) were included in final analysis based on the eligibility criteria. Based on the medians, the cut-off values of MLR, NLR, and PLR were set as 0.223, 2.22, and 124.4, respectively, and patients were divided into low- and high-level groups for further analysis. The clinicopathological characteristics of the overall and ratio-specific patient groups are presented in Table 1. The median follow-up month was 72 (interquartile, 35-98).

**Table 1:** Patients’ characteristics

Parameter	Overall	MLR ≤ 0.223	MLR > 0.223	NLR ≤ 2.22	NLR > 2.22	PLR ≤ 124.4	PLR > 124.4
*n*	611	305	306	305	306	306	305
*Gender*							
Female	123 (20)	72 (24)	51 (17)	65 (21)	58 (19)	53 (17)	70 (23)
Male	488 (80)	233 (76)	255 (83)	240 (79)	248 (81)	253 (83)	235 (77)
*Age (years)*	62 ± 10; 63 (30-89)	62 ± 9; 62 (30-80)	63 ± 10; 64 (30-89)	62 ± 9; 63 (30-80)	62 ± 10; 63 (30-89)	63 ± 9; 63 (30-80)	62 ± 10; 63 (33-89)
*Age group*							
≤ 63 years	321 (53)	172 (56)	149 (49)	163 (53)	158 (52)	162 (53)	159 (52)
> 63 years	290 (47)	133 (44)	157 (51)	142 (47)	148 (48)	144 (47)	146 (48)
*Siewert classification*							
Type II	390 (64)	196 (64)	194 (63)	202 (66)	188 (61)	209 (68)	181 (59)
Type III	221 (36)	109 (36)	112 (37)	103 (34)	118 (39)	97 (32)	124 (41)
*Tumor length (cm)*	5 ± 3; 5 (1-25)	5 ± 3; 5 (1-25)	6 ± 3; 5 (1-20)	5 ± 3; 5 (1-25)	6 ± 3; 5 (1-20)	5 ± 3; 4 (1-17)	6 ± 3; 5 (1-25)
*Tumor length group*							
≤ 5 cm	360 (59)	198 (65)	162 (53)	195 (64)	165 (54)	207 (68)	153 (50)
> 5 cm	251 (41)	107 (35)	144 (47)	110 (36)	141 (46)	99 (32)	152 (50)
*Nerve invasion*							
No	590 (97)	296 (97)	294 (96)	294 (96)	296 (97)	296 (97)	294 (96)
Yes	21 (3)	9 (3)	12 (4)	11 (4)	10 (3)	10 (3)	11 (4)
*Vessel invasion*							
No	506 (83)	258 (85)	248 (81)	247 (81)	259 (85)	255 (83)	251 (82)
Yes	107 (17)	47 (15)	58 (19)	58 (19)	47 (15)	51 (17)	54 (18)
*pT stage*							
1a	27 (4)	12 (4)	14 (5)	12 (4)	15 (5)	14 (5)	13 (4)
1b	37 (6)	25 (8)	12 (4)	22 (7)	15 (5)	25 (8)	12 (4)
2	60 (10)	28 (9)	32 (10)	28 (9)	32 (10)	32 (10)	28 (9)
3	4 (1)	1 (0)	3 (1)	2 (1)	2 (1)	2 (1)	2 (1)
4a	361 (59)	189 (62)	172 (56)	191 (63)	170 (56)	182 (59)	179 (59)
4b	122 (20)	49 (16)	73 (24)	50 (16)	72 (24)	51 (17)	71 (23)
*pN stage*							
0	214 (35)	114 (37)	100 (33)	114 (37)	100 (33)	118 (39)	96 (31)
1	37 (6)	17 (6)	20 (7)	8 (3)	29 (9)	11 (4)	26 (9)
2	147 (24)	75 (25)	72 (24)	84 (28)	63 (21)	79 (26)	68 (22)
3	213 (35)	99 (32)	114 (37)	99 (32)	114 (37)	98 (32)	115 (38)
*pTNM stage*							
IA	59 (10)	33 (11)	26 (9)	31 (10)	28 (9)	35 (11)	24 (8)
IB	39 (6)	17 (6)	22 (7)	18 (6)	21 (7)	19 (6)	20 (7)
IIA	9 (1)	7 (2)	2 (1)	5 (2)	4 (1)	5 (2)	4 (1)
IIB	104 (17)	60 (20)	44 (14)	59 (19)	45 (15)	63 (21)	41 (13)
IIIA	33 (5)	14 (5)	19 (6)	10 (3)	23 (8)	15 (5)	18 (6)
IIIB	140 (23)	68 (22)	72 (24)	77 (25)	63 (21)	65 (21)	71 (25)
IIIC	227 (37)	106 (35)	121 (40)	105 (34)	122 (40)	104 (34)	123 (40)
*Total leucocyte count (× 10^9^/L)*	5.8 ± 1.9; 5.5 (1.3-19.0)	5.7 ± 1.6; 5.6 (2.6-14.4)	6.0 ± 2.2; 5.6 (1.3-19.0)	5.3 ± 1.4; 5.2 (1.3-9.5)	6.4 ± 2.2; 6.0 (2.1-19.0)	6.0 ± 1.8; 5.8 (2.0-15.5)	5.6 ± 2.1; 5.3 (1.3-19.0)
*Lymphocyte count (× 10^9^/L)*	1.6 ± 0.5; 1.5 (0.4-3.9)	1.8 ± 0.5; 1.8 (0.8-3.9)	1.3 ± 0.4; 1.3 (0.4-2.8)	1.8 ± 0.5; 1.8 (0.4-3.9)	1.3 ± 0.4; 1.3 (0.4-3.0)	1.9 ± 0.5; 1.8 (0.6-3.9)	1.3 ± 0.4; 1.3 (0.4-2.6)
*Monocyte count (× 10^9^/L)*	0.4 ± 0.1; 0.3 (0.1-1.0)	0.3 ± 0.1; 0.3 (0.1-0.6)	0.4 ± 0.1; 0.4 (0.1-1.0)	0.3 ± 0.1; 0.3 (0.1-1.0)	0.4 ± 0.1; 0.4 (0.1-0.9)	0.4 ± 0.1; 0.4 (0.1-0.9)	0.3 ± 0.1; 0.3 (0.1-1.0)
*Neutrophil count (× 10^9^/L)*	3.7 ± 1.8; 3.4 (0.7-17.6)	3.3 ± 1.2; 3.2 (0.8-10.4)	4.1 ± 2.1; 3.6 (0.7-17.6)	2.9 ± 0.9; 2.9 (0.7-5.3)	4.5 ± 2.0; 4.1 (1.3-17.6)	3.6 ± 1.5; 3.4 (0.8-13.8)	3.8 ± 2.0; 3.4 (0.7-17.6)
*Platelet count (× 10^9^/L)*	197 ± 73; 187 (32-761)	196 ± 71; 186 (65-670)	198 ± 76; 187 (32-761)	196 ± 65; 187 (32-405)	199 ± 81; 187 (64-761)	163 ± 52; 161 (32-326)	231 ± 76; 222 (91-761)
*MLR*	0.25 ± 0.13; 0.22 (0.07-1.30)	0.17 ± 0.03; 0.17 (0.07-0.22)	0.33 ± 0.13; 0.30 (0.22-1.30)	0.20 ± 0.07; 0.18 (0.07-0.72)	0.31 ± 0.14; 0.28 (0.07-1.30)	0.21 ± 0.09; 0.19 (0.07-0.72)	0.29 ± 0.15; 0.26 (0.10-1.30)
*NLR*	2.2 ± 2.7; 2.5 (0.4-28.6)	1.9 ± 0.7; 1.7 (0.4-4.7)	3.5 ± 3.3; 2.8 (0.7-28.6)	1.6 ± 0.4; 1.7 (0.4-2.2)	3.8 ± 3.2; 3.0 (2.2-28.6)	2.1 ± 1.1; 1.9 (0.4-15.7)	3.4 ± 3.3; 2.7 (0.7-28.6)
*PLR*	124 ± 139; 88 (35-1585)	113 ± 47; 104 (35-328)	165 ± 109; 151 (44-1585)	114 ± 49; 105 (35-398)	165 ± 108; 149 (43-1585)	90 ± 21; 92 (35-124)	189 ± 100; 171 (125-1585)

Enumeration data are shown as n (percentage [%]), and measurement data as mean ± standard deviation; median (range).

Abbreviations: MLR, monocyte-lymphcyte ratio; NLR, neutrophil-lymphocyte ratio; PLR, platelet-lymphocyte ratio.

### MLR, NLR and PLR in relation to clinicopathological features

The association is shown in Table 2. In overall patients, MLR was significantly higher in male patients and those older than 63 years; NLR was insignificantly higher in those with type III tumors; and PLR was significantly elevated in people with type III tumors. In patients with type II tumors, MLR was insignificantly higher in those > 63 years, while no significant observations were observed for NLR and PLR.

**Table 2:** Association of preoperative monocyte-lymphocyte, neutrophil-lymphocyte and platelet-lymphocyte ratios with clinicopathological parameters in overall patients and those with Siewert types II and III adenocarcinoma of esophagogastric junction

Parameter		MLR^a^			NLR^b^			PLRc		
		*Overall*	*Siewert type II*	*Siewert type III*	*Overall*	*Siewert type II*	*Siewert type III*	*Overall*	*Siewert type II*	*Siewert type III*
*Gender*	Female	1 (reference)	1 (reference)	1 (reference)	1 (reference)	1 (reference)	1 (reference)	1 (reference)	1 (reference)	1 (reference)
	Male	1.51 (1.00-2.27)	1.38 (0.80-2.38)	1.69 (0.89-3.22)	1.16 (0.77-1.73)	1.23 (0.71-2.11)	1.10 (0.58-2.09)	0.70 (0.46-1.05)	0.66 (0.38-1.14)	0.72 (0.38-1.37)
*Age group*	≤ 63 years	1 (reference)	1 (reference)	1 (reference)	1 (reference)	1 (reference)	1 (reference)	1 (reference)	1 (reference)	1 (reference)
	> 63 years	1.38 (1.00-1.91)	1.36 (0.91-2.04)	1.45 (0.83-2.53)	1.13 (0.82-1.57)	1.14 (0.76-1.71)	1.16 (0.66-2.02)	1.09 (0.79-1.52)	1.07 (0.71-1.60)	1.15 (0.66-2.00)
*Siewert classification*	Type II	1 (reference)	NA	NA	1 (reference)	NA	NA	1 (reference)	NA	NA
	Type III	1.12 (0.79-1.57)	NA	NA	1.28 (0.92-1.80)	NA	NA	1.48 (1.06-2.09)	NA	NA
*Nerve invasion*	No	1 (reference)	1 (reference)	1 (reference)	1 (reference)	1 (reference)	1 (reference)	1 (reference)	1 (reference)	1 (reference)
	Yes	1.30 (0.53-3.22)	2.21 (0.55-8.89)	0.93 (0.26-3.33)	1.01 (0.41-2.48)	1.87 (0.51-6.90)	0.53 (0.14-1.93)	1.10 (0.45-2.71)	1.25 (0.35-4.50)	0.98 (0.28-3.52)
*Vessel invasion*	No	1 (reference)	1 (reference)	1 (reference)	1 (reference)	1 (reference)	1 (reference)	1 (reference)	1 (reference)	1 (reference)
	Yes	1.15 (0.74-1.78)	1.38 (0.79-2.42)	0.83 (0.40-1.74)	0.71 (0.46-1.11)	0.64 (0.36-1.12)	0.85 (0.40-1.78)	0.94 (0.60-1.46)	0.91 (0.52-1.59)	0.98 (0.47-2.03)
*pTNM stage*	I	1 (reference)	1 (reference)	1 (reference)	1 (reference)	1 (reference)	1 (reference)	1 (reference)	1 (reference)	1 (reference)
	II	0.73 (0.42-1.26)	0.60 (0.30-1.20)	0.98 (0.39-2.51)	0.76 (0.44-1.32)	0.58 (0.29-1.17)	1.45 (0.57-3.71)	0.74 (0.43-1.30)	0.77 (0.38-1.57)	0.66 (0.26-1.67)
	III	1.06 (0.67-1.69)	0.99 (0.56-1.72)	1.23 (0.54-2.77)	1.06 (0.67-1.68)	0.82 (0.47-1.44)	1.90 (0.83-4.33)	1.34 (0.85-2.14)	1.52 (0.86-2.67)	1.05 (0.46-2.37)

Risk ratios (RRs) of high to low level of each ratio for overall patients with adenocarcinoma of esophagogastric junction, those with type II tumors, and those with stage III cancers by clinicopathological characteristics are shown as point estimate (95% confidence interval) with respect to a reference group (defined as 1). RRs were calculated using the multiple Logistic regression model adjusting gender, age group, tumor location, nerve and vessel invasion, nodule formation, and pTNM stage. Statistically significant point estimates are shown in bold. ^a^MLR > 0.223 vs. MLR ≤ 0.223; ^b^NLR > 2.22 vs. NLR ≤ 2.22; ^c^PLR > 124.4 vs. PLR ≤ 124.4

Abbreviations: MLR, monocyte-lymphocyte ratio; NLR, neutrophil-lymphocyte ratio; PLR, platelet-lymphocyte ratio; NA, not applicable.

### MLR, NLR and PLR in relation to CSS

Based on univariate analysis, advanced pTNM stage, higher MLR and NLR were significantly associated with a higher death risk; after incorporating various factors in the Cox proportional hazards model-based multivariate analysis, pTNM stage was revealed to be an independent prognostic factor, and MLR tended to be independently associated with CSS, although insignificantly. No associated was found for PLR. (Table 3)

**Table 3:** Univariate and multivariate analyses of monocyte-lymphocyte, neutrophil-lymphocyte and platelet-lymphocyte ratios in relation to adenocarcinoma of esophagogastric junction-specific survival

Parameter	Univariate analysis	Multivariate analysis
Gender	1.11 (0.82-1.49)	1.07 (0.79-1.45)
Age (years)	1.01 (0.99-1.02)	1.01 (0.99-1.02)
Siewert classification	1.07 (0.84-1.37)	1.11 (0.86-1.43)
Nerve invasion	0.96 (0.48-1.95)	0.94 (0.46-1.92)
Vessel invasion	1.09 (0.80-1.49)	0.82 (0.60-1.12)
pTNM stage	**1.98** (1.63-2.42)	**1.96** (1.60-2.41)
Monocyte-lymphocyte ratio	**3.00** (1.36-6.63)	2.68 (0.85-8.43)a
Neutrophil-lymphocyte ratio	**1.04** (1.00-1.08)	1.00 (0.94-1.07)
Platelet-lymphocyte ratio	1.00 (1.00-1.00)	1.00 (1.00-1.00)

Data are shown as hazard ratio (95% confidence interval). Statistically significant point estimates are shown in bold.

Continuous ratios were applied.

^a^P = 0.092

Stratified analyses were further conducted. Overall, MLR was significantly associated with a higher postsurgical death risk in male patients, those ≤ 63 years, those with type II tumors, and a tendency was observed in those without vessel invasion and those with stage III tumors; NLR was significantly associated with a higher death risk in female patients, those with type III tumors, and those with vessel invasion, and a tendency was detected in those > 63 years, and those with stage III tumors. In Siewert type II tumors, tendencies were observed in those ≤ 63 years for MLR. In type III AEG, significant associations were detected in male patients, those with type III tumors, those with vessel invasion, and those with stage III tumors for MLR, and in both genders, those > 63 years, those with and without vessel involvement, and those with stage III tumors for NLR. For PLR, a significant association was only observed in older people with type III cancers. (Table 4)

**Table 4:** MLR, NLR and PLR in relation to AEG-specific survival in various stratifications of overall patients and those with types II and III AEG

Parameter	MLR^a^			NLR^b^			PLRc		
	Overall	Siewert type II	Siewert type III	Overall	Siewert type II	Siewert type III	Overall	Siewert type II	Siewert type III
**Used no.**	611	390	221	611	390	221	611	390	221
**Comprehensive**	1.26 (0.99-1.59)	1.06 (0.79-1.43)	**1.69** (1.13-2.54)	1.26 (1.00-1.60)	1.03 (0.77-1.39)	**1.84** (1.22-2.78)	0.99 (0.78-1.25)	0.87 (0.64-1.17)	1.27 (0.85-1.91)
**Gender**									
Female	1.03 (0.59-1.80)	0.59 (0.27-1.33)	1.42 (0.61-3.29)	**1.96** (1.08-3.53)	1.41 (0.64-3.10)	**2.93** (1.03-8.31)	1.03 (0.58-1.80)	0.79 (0.38-1.67)	1.25 (0.52-3.03)
Male	1.33 (1.02-1.73)	1.18 (0.85-1.63)	1.71 (1.07-2.75)	1.16 (0.90-1.51)	0.98 (0.71-1.35)	**1.69** (1.06-2.70)	0.98 (0.75-1.27)	0.86 (0.62-1.19)	1.33 (0.84-2.11)
**Age group**									
≤ 63 years	1.55 (1.11-2.16)	1.54 (1.00-2.37)	1.57 (0.93-2.67)	1.17 (0.84-1.63)	1.10 (0.71-1.69)	1.28 (0.75-2.17)	0.94 (0.68-1.31)	0.92 (0.60-1.42)	1.03 (0.61-1.74)
> 63 years	0.98 (0.70-1.37)	0.74 (0.49-1.12)	1.74 (0.91-3.33)	1.32 (0.94-1.85)	0.93 (0.62-1.41)	**2.94** (1.46-5.89)	1.08 (0.77-1.53)	0.84 (0.56-1.27)	**1.99** (1.02-3.87)
**Siewert classification**									
Type II	1.06 (0.79-1.43)	1.06 (0.79-1.43)	NA	1.03 (0.77-1.39)	1.03 (0.77-1.39)	NA	0.87 (0.64-1.17)	0.87 (0.64-1.17)	NA
Type III	**1.69** (1.13-2.54)	NA	**1.69** (1.13-2.54)	**1.84** (1.22-2.78)	NA	**1.84** (1.22-2.78)	1.27 (0.85-1.91)	NA	1.27 (0.85-1.91)
**Vessel invasion**									
No	1.23 (0.95-1.60)	1.11 (0.80-1.53)	1.49 (0.96-2.32)	1.17 (0.91-1.52)	0.96 (0.69-1.33)	**1.70** (1.09-2.67)	0.90 (0.67-1.17)	0.80 (0.57-1.11)	1.12 (0.72-1.74)
Yes	1.49 (0.80-2.78)	0.95 (0.46-1.97)	**3.50** (1.06-11.51)	**2.08** (1.14-3.80)	1.58 (0.75-3.32)	**5.11** (1.24-21.16)	1.60 (0.90-2.84)	1.26 (0.62-2.55)	2.94 (0.90-9.64)
**pTNM stage**									
I	1.44 (0.52-3.95)	0.66 (0.20-2.10)	NA	0.67 (0.25-1.82)	0.82 (0.26-2.58)	0.32 (0.03-3.72)	1.60 (0.58-4.43)	1.60 (0.51-5.02)	1.03 (0.08-12.78)
II	1.13 (0.62-2.08)	1.06 (0.51-2.23)	1.00 (0.33-2.99)	1.58 (0.87-2.87)	1.75 (0.85-3.63)	1.27 (0.45-3.62)	1.35 (0.75-2.44)	1.16 (0.55-2.45)	1.64 (0.57-4.68)
III	1.27 (0.97-1.67)	1.10 (0.78-1.55)	**1.58** (1.01-2.47)	1.26 (0.96-1.65)	0.95 (0.68-1.34)	**2.03** (1.27-3.25)	0.92 (0.70-1.20)	0.79 (0.56-1.10)	1.18 (0.75-1.84)

Hazard ratios (HRs) of high versus low level of each ratio are presented as point estimate (95% confidence interval) after adjustment of gender, age group, tumor position, vessel invasion, and pTNM stage in each stratification by clinicopathological parameters of the overall patients, patients with type II tumors, and those with type III cancers. HRs were calculated using the multiple Cox regression model with adjustment, and are statistically significant when shown in bold. ^a^MLR > 0.223 vs. MLR ≤ 0.223; ^b^NLR > 2.22 vs. NLR ≤ 2.22; ^x^PLR > 124.4 vs. PLR ≤ 124.4 Abbreviations: MLR, monocyte-lymphocyte ratio; NLR, neutrophil-lymphocyte ratio; PLR, platelet-lymphocyte ratio; NA, not applicable.

**Figure 1:**
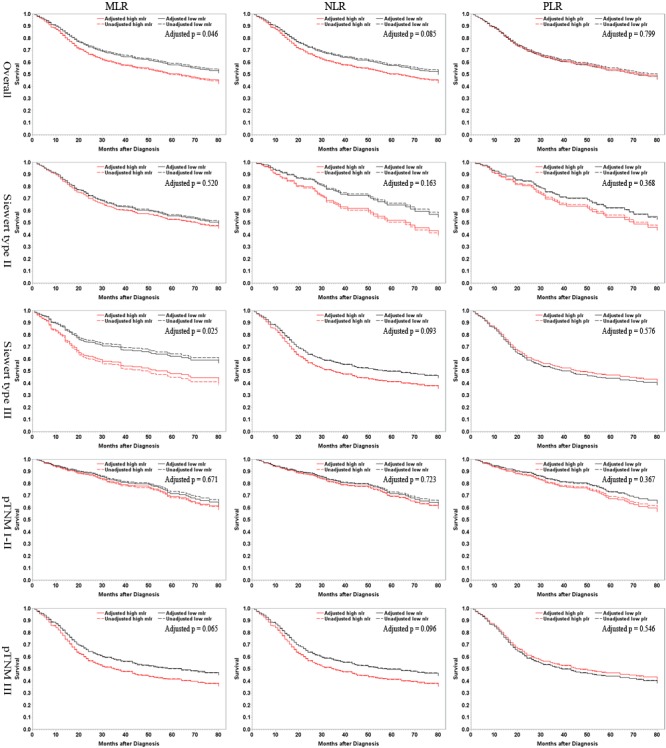
High- *versus* low-level MLR, NLR and PLR in relation to AEG-specific survival among overall patients and in tumor position- and stage-specific subgroups Dashed lines are for unadjusted results, and solid lines represent adjusted outcomes. Factors for adjustment include gender, age, tumor position, stage, and vessel invasion. MLR, monocyte-lymphocyte ratio; NLR, neutrophil-lymphocyte ratio; PLR, platelet-lymphocyte ratio.

Accordingly and consistently, the unadjusted and adjusted survival curves for the overall, tumor position- and stage- specific patient groups are show in Figure 1, and the periodical survival rates in the low- and high-level arms of the 3 ratios are listed in Table 5. Upon 5-year period, in the overall patients, 58% of patients survived in the low-MLR group compared to 50% in the high-level group, 57% survived in the low-NLR group compared to 52% in the high-level group, and 55% survived in the low-PLR group compared to 54% in the high-level group; in those with type II tumors, 56% of people survived in the low-MLR group compared to 53% in the high-level group, 53% survived in the low-NLR group compared to 56% in the high-level group, and 54% survived in the low-PLR group compared to 55% in the high-level group; in those with type III tumors, 62% of individuals survived in the low-MLR group compared to 46% in the high-level group, 64% survived in the low-NLR group compared to 44% in the high-level group, and 54% survived in the low-PLR group compared to 52% in the high-level group.

**Table 5:** Unadjusted survival rates for overall patients and those with types II and III AEG

Time	Level	MLR^a^			NLR^b^			PLR^c^		
		Overall	Siewert type II	Siewert type III	Overall	Siewert type II	Siewert type III	Overall	Siewert type II	Siewert type III
3-month	Low	98.3 (96.0-99.3)	99.0 (95.9-99.7)	97.1 (91.2-99.1)	98.7 (96.5-99.5)	98.5 (95.5-99.5)	99.0 (93.3-99.9)	98.4 (96.1-99.3)	98.1 (95.0-99.3)	99.0 (92.9-99.9)
	High	95.9 (93.0-97.6)	96.5 (92.8-98.3)	94.9 (89.0-97.7)	97.7 (95.3-98.9)	98.9 (95.8-98.7)	95.8 (90.1-98.2)	98.0 (95.7-99.1)	99.4 (96.1-99.9)	96.0 (90.6-98.3)
6-month	Low	95.9 (92.9-97.7)	97.4 (93.8-98.9)	93.2 (86.3-96.7)	96.4 (93.6-98.0)	96.5 (92.5-99.3)	96.1 (90.0-98.5)	95.1 (92.0-97.0)	94.7 (90.7-97.1)	95.9 (89.4-98.4)
	High	91.5 (87.8-94.1)	90.5 (85.4-93.8)	93.2 (86.9-96.6)	93.5 (90.1-95.7)	94.1 (89.7-96.7)	92.4 (85.9-96.0)	94.8 (91.6-96.8)	96.1 (92.1-98.1)	92.7 (86.5-96.2)
12-month	Low	88.4 (84.2-91.6)	90.1 (84.8-93.5)	85.4 (77.0-91.0)	91.1 (87.4-93.8)	90.6 (85.7-93.9)	92.2 (85.1-96.0)	87.9 (83.7-91.1)	88.5 (83.4-92.2)	86.6 (78.0-92.0)
	High	83.0 (78.4-86.7)	83.9 (78.0-88.3)	81.4 (73.1-87.3)	83.7 (79.0-87.4)	87.2 (81.6-91.3)	78.0 (69.4-86.4)	86.9 (82.6-90.2)	89.5 (84.0-93.2)	83.1 (75.2-88.6)
24-month	Low	73.5 (68.0-78.1)	72.8 (65.9-78.5)	74.8 (65.2-82.1)	74.4 (69.1-78.9)	72.8 (66.1-78.4)	77.7 (68.3-84.6)	72.9 (67.5-77.5)	73.7 (67.2-79.1)	71.1 (61.0-79.1)
	High	66.2 (60.8-71.1)	69.8 (63.0-75.7)	60.2 (50.8-68.4)	67.6 (62.1-72.6)	73.4 (66.5-79.1)	58.5 (49.0-66.8)	69.2 (63.7-74.0)	72.4 (65.2-78.3)	64.5 (55.4-72.2)
36-month	Low	65.6 (60.0-70.8)	62.8 (59.6-65.2)	70.9 (61.1-78.6)	65.6 (60.0-70.6)	61.4 (54.3-67.7)	73.8 (64.2-81.2)	64.4 (58.7-69.5)	63.2 (56.2-69.3)	67.0 (56.7-75.4)
	High	59.9 (54.3-65.1)	62.3 (55.2-68.6)	55.9 (46.5-64.3)	60.8 (55.1-66.0)	65.4 (58.2-71.7)	53.4 (44.0-61.9)	62.0 (56.3-67.1)	63.5 (56.1-70.1)	59.7 (50.5-67.7)
60-month	Low	58.4 (52.2-64.1)	56.2 (48.6-63.1)	62.3 (50.5-72.1)	56.7 (50.5-62.5)	53.4 (65.9-40.3)	64.2 (52.8-73.5)	54.5 (48.3-60.4)	54.2 (46.9-60.9)	53.8 (40.3-65.6)
	High	50.1 (44.0-55.8)	52.9 (45.2-60.1)	45.6 (35.6-55.0)	51.5 (45.3-57.3)	56.0 (48.2-63.0)	44.3 (34.3-53.8)	53.6 (47.5-59.3)	55.1 (47.1-62.4)	51.7 (42.1-60.4)

Data are shown as point estimate (95% confidence interval).

^a^Low level: ≤ 0.223, high level: > 0.223; ^b^low level: ≤ 2.22, high level: > 2.22; ^c^low level: PLR ≤ 124.4, high level: PLR > 124.4

Abbreviations: MLR, monocyte-lymphocyte ratio; NLR, neutrophil-lymphocyte ratio; PLR, platelet-lymphocyte ratio.

## DISCUSSION

Systemic inflammation correlates with tumor progression. Various peripheral inflammatory markers including MLR, NLR and PLR and their prediction of clinical outcomes in diverse tumor entities have been uncovered [9]. These biomarkers also predictive of responses to immunotherapies remain an area of unmet need. In this study, we examined a large prospective cohort of patients with type II/III AEG, and investigated the clinicopathological and prognostic significances of preoperative MLR, NLR, and PLR as markers predicting the outcomes after radical gastrectomy. To the best of our knowledge, few previous studies [15, 16] assessing the ratios in AEG have been reported, and both found a significant association of pretreatment NLR with prognosis in AEG patients. The prognostic significances of the ratios were also reported in gastric cancer [17] and esophageal cancer [18–21], which however might differ greatly in etiology, origin, tumor biology, histology, and prognosis from type II/III AEG, which might be considered as an independent cancer type [6, 22], making the findings in the 2 cancer entities potentially inapplicable in AEG. Regarding NLR in AEG, our results were consistent with the previous overall findings [15, 16], and we had more detailed subgroup analyses with longer follow-up period. Besides, we further investigated MLR and PLR, and showed that both MLR and NLR were prognosis-indicative in type III but not type II AEG, while PLR had very limited significance.

Lymphocytes play essential roles in systemic inflammatory response to tumorous diseases, including the inhibition of tumor cell proliferation and migration [23]. Lymphocytes infiltrating to tumor microenvironment could trigger immunological antitumor reactions. Lymphocyte interaction with other inflammation cells could be essential in the anti-tumor reaction of the immune system (*e.g*., by inducing tumor cell apoptosis) [24]. Decreased lymphocyte count could reflect insufficient immunologic reaction to tumor, with enhanced tumor proliferation and metastasis [25]. Lymphopenia is a powerful predictor of clinical outcomes in hematologic and solid malignancies [26]. Lymphopenia prior to initiation of systematic treatment is a poor risk feature in cancer patients who have been treated with neoadjuvant therapy [27]. Lymphopenia might impair the efficacy of immune system by impairing antibody-dependent cell-mediated cytotoxicity, which plays important roles in slowing or preventing tumor progression and distant metastasis, due to lack of effector cells. Due to its definite role in tumor immunity, lymphocyte count was applied as the standard in the 3 ratios.

The role of monocytes/macrophages in tumor progression is essential [28]. There is increasing evidence that tumor-associated macrophages (TAMs), which are derived from circulating monocytes, suppress the host immune system and promote tumor angiogenesis, proliferation, migration, and metastasis [29]. There is a pro-tumorous potential of monocytes *via* formation of different malignancy-promoting macrophage phenotypes. High peripheral absolute monocyte count indicates formation of TAMs and elevated tumor burden in patients. Accordingly, peripheral blood monocytosis is an adverse prognostic factor in various tumors [30, 31]. MLR might be well reflective of responsiveness of the host immune system and a microenvironment surrogate marker of tumor burden. In various hematologic malignancies [32, 33] and solid tumors [31, 34–40] including lung cancer, bladder cancer, breast cancer, oropharyngeal cancer, pancreatic adenocarcinoma, colorectal cancer and renal cell carcinoma, high pre-treatment peripheral MLR level is significantly unfavorable and represents a useful outcome-predictive marker. The prognostic values of MLR in AEG patients remain uncertain. Based on our prospective cohort, MLR was higher in male patients and older individuals; it tended to be an independent prognostic factor, and increased MLR was associated with poorer CSS in male patients, younger people, those with type III tumors, and those with stage III tumors. MLR thus provides an easily-available and low-cost prognostic biomarker. Interestingly and notably, there existed gender-, age-, tumor position- and stage-specific association between MLR and survival. Type III AEG might be more similar to gastric cancer, for which MLR has been revealed to be prognostically significant [39]. Immunoediting occurs with tumor progression [41]. The observation that MLR tended to be prognosis-predictive in stage III tumors might be explained by the fact that pro-tumorous immune cells including Mψ are activated at advanced stages to facilitate the latter metastatic progression. The gender and age discrepancies might be determined by physiological characteristics.

High neutrophil proportion in tumor stroma is associated with poor prognoses [42, 43]. Neutrophils enhance tumor progression by inducting mutation of cancer suppressive genes, secreting enzymes and cytokines to facilitate malignant cell proliferation and metastasis, and promoting tumor angiogenesis [44, 45]. NLR has been validated as a prognostic biomarker in patients in various cancers, identifying patients whose tumors are generating inflammatory responses [46, 47]. There is scope for further investigation of NLR as a predictive biomarker of response to immunotherapies, particularly with immune checkpoint-targeting drugs like PD-1/PDL-1 and CTLA-4 targeting antibodies [48, 49], and the utility of NLR normalization within treatment. We observed that NLR was higher in patients with type II AEG. It was prognosis-predictive in females, older patients, those with type III tumors, and those with stage III tumors. The phenomenon that the significance occurred with the advancement of tumor stage could be partly explained by that at later stages, tumor cells secrete chemokines guiding neutrophils into tumor microenvironment, with peripheral neutrophil proliferation activated, which could conversely suppress lymphocytes [50]. In gastric cancer, correlation between NLR and tumor progression was also reported [11].

The only significant findings concerning PLR were that it was higher in patients with type III tumors, and that its elevation was associated with worse CSS only in older patients with type III AEG, although its prognostic significance has been reported in various other cancer entities [51–53]. The difference might partly lie in the fact that many other researches did not adjust confounding factors sufficiently like the way we did, which could reveal the true association.

The strengths of this study include but are not limited to the prospective nature, the large sample size, the long follow-up period, the application of CSS in survival analysis, and the appropriate and rigorous methodology. However, there are some potential limitations. Firstly, other potential confounding factors including comorbidities that might affect the blood cell count were not considered. Besides, there might be other reasonable cut-off levels for each ratio.

Together, this large prospective evidence showed that MLR and NLR might be potential prognostic factors in patients with non-metastatic Siewert type II/III AEG, especially in those with type III and stage III tumors, while PLR had limited significance.
